# Evaluation of Physiological State of Pen Shell *Pinna nobilis* (Linnaeus, 1758) by a Non-Invasive Heart Rate Recording under Short-Term Hyposalinity Test

**DOI:** 10.3390/mi13091549

**Published:** 2022-09-18

**Authors:** Rajko Martinović, Danijela Joksimović, José Rafael García-March, Nardo Vicente, Zoran Gačić

**Affiliations:** 1 Institute of Marine Biology, University of Montenegro, Put I Bokeljske Brigade 68, 85330 Kotor, Montenegro; 2 Instituto de Investigación en Medio Ambiente y Ciencia Marina, Universidad Católica de Valencia, 03710 Calpe, Alicante, Spain; 3 Institut Océanographique Paul Ricard, Ile des Embiez, 83140 Six-Fours-les-Plages, France; 4 Institut Méditerranéen de Biodiversité et Ecologie Marine et Continentale (IMBE), Aix–Marseille Université, CNRS, IRD, Avignon Université, 74 Rue Louis Pasteur, 84029 Avignon, France; 5 Institute for Multidisciplinary Research, University of Belgrade, Kneza Višeslava 1, 11000 Belgrade, Serbia

**Keywords:** *Pinna nobilis*, pen shell, heart rate, mussels, physiology, hyposalinity

## Abstract

A non-invasive laser fiber-optic method based on infrared sensors for heart rate (Hr) recording was applied to assess the physiological condition of *Pinna nobilis*. During 2017, the specimens of *P. nobilis* were sampled at three sites within the Boka Kotorska Bay, Montenegro and used for ex situ experiments with short-term reduction/restoration of ambient salinity to evaluate their physiological adaptive capacity based on heart rate recovery time (T_rec_). Mean T_rec_ for specimens from Sv. Nedjelja (reference site), Dobrota and Sv. Stasije were 72 ± 3, 91 ± 7 and 117 ± 15 min, while the coefficients of variation (CV) were 0.12, 0.13 and 0.17, respectively. Resting heart rate (Hr_rest_) and T_rec_ showed statistically significant differences between the groups of mussels from Dobrota and Sv. Stasije in comparison to the reference site. Statistically significant correlations were observed between T_rec_ and shell length/width, which was not the case in comparison between Hr_rest_ and shell length/width. The lower adaptive capacity within the *P. nobilis* specimens from Dobrota and Sv. Stasije in comparison to the reference site could occur due to stress induced by deterioration of environmental conditions, which could have led to impairment of the physiological state of the mussels evaluated by Hr. All the specimens of *P. nobilis* survived the experimental treatments; afterwards, they were successfully transplanted at the Dobrota site. The experimental unit with sensor technology applied in this study can provide Hr recording in real time and could have an application in monitoring the physiological/health state of *P. nobilis* individuals maintained in aquaria.

## 1. Introduction

Current trends of global climate change, such as extreme draughts or strong rainfall, contributed to more intensive fluctuations in coastal salinity, influencing marine organisms [1,2]. Estuarine bivalves are particularly sensitive to hyposalinity conditions due to activity reduction, and high-energy demands to maintain ion homeostasis and avoid irreversible cell damage [3,4]. However, mussels have interesting physiological mechanisms of adaptation to variable salinity [5]. The heart rate (Hr) of marine mussels as a physiological biomarker measured by non-invasive infrared-based sensors [6] proved to be a reliable indicator of environmental salinity changes [7,8,9,10]. Moreover, a similar methodology for Hr registration, the fiber-optic method [11], has an application in the assessment of aquatic ecosystem health by investigation of a mussel’s physiological condition. This was achieved by ex situ standardized test based on the calculation of heart rate recovery time (T_rec_) after rapid change in ambient water salinity as a stress stimulus [12]. A shorter T_rec_ in mussels from the clean site in comparison with the polluted one as an indication of higher adaptive capacity and good health condition was the main hypothesis, evaluated in many studies [13,14,15,16,17,18].

The pen shell *Pinna nobilis* (Linnaeus, 1758) is an endemic Mediterranean bivalve; its large shell can reach 120 cm of antero–posterior length [19], burrowed in marine sediment at depths ranging from 0.5 to 60 m [20]. *P. nobilis* became endangered due to fishing, habitat degradation and marine pollution [21]. Moreover, since late 2016, disease amongst *P. nobilis* caused by the protozoan parasite *Haplosporidium pinnae* sp. nov. [22] and/or mycobacterial disease [23] has been occurring, starting from Spain and spreading all over the Mediterranean, with a mortality rate of up to 100% [24,25]. Consequently, *P. nobilis* is marked as Critically Endangered in the IUCN Red List of Threatened Species [26]. The remaining populations of *P. nobilis* on the western Mediterranean coast of Spain and France have survived under the fluctuating environment of lagoons and estuarine [27,28,29,30]. The Boka Kotorska Bay (Adriatic Sea) is exposed to many sources of freshwater inputs which can strongly modify the pattern of the seawater temperature, salinity and currents [31]. Besides the physiological studies on pen shell gaping activity [32,33,34], respiration rates [35] and osmoregulation [36], there is a lack of studies on the influence of environmental parameters on *P. nobilis* Hr.

Considering all of the aforementioned, the main aim of this study was to assess *P. nobilis’* health condition in the Boka Kotorska Bay by a non-invasive fiber-optic method for Hr recording of mussels, under short-term ex situ salinity reduction test. In addition, the aim was to compare the Hr_rest_ and T_rec_ of shells with different sizes. Moreover, one of the purposes was the transplantation of *P. nobilis* specimens from a more to a less dense population toward the goal of species’ protection. The study was carried out during 2017 as part of the PinnaSpot project: the study, protection and possible breeding of the pen shell (*Pinna nobilis*) in the Boka Kotorska Bay, two years before the occurrence of the mass mortality event (MME) of *P. nobilis* in the Adriatic Sea [37].

## 2. Materials and Methods

### 2.1. Specimen Collection and Relocation

**Figure 1:**
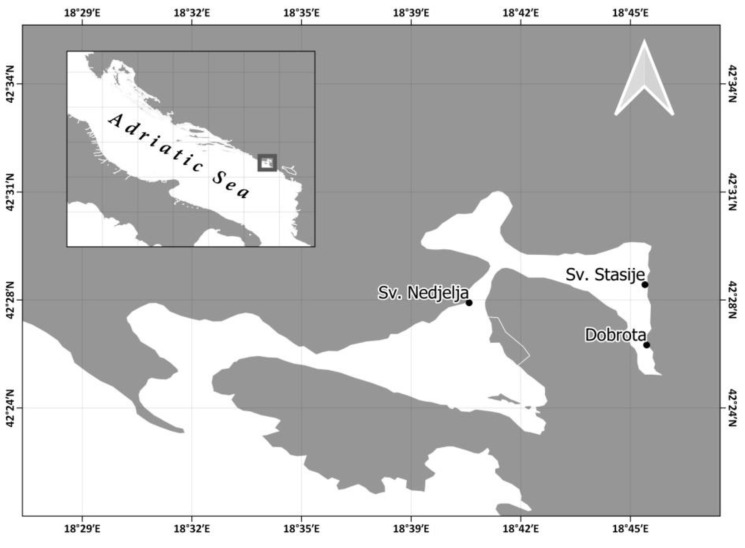
Sampling sites in the Boka Kotorska Bay, Montenegro.

Since *P. nobilis* is endangered and protected under ANNEX II of the Barcelona Convention and ANNEX IV of the EC Habitats Directive 92/43/EEC, before the implementation of any activities envisaged by the PinnaSpot project, including the present study, a confirmation letter from the relevant state authorities in Montenegro was obtained (enclosed). During the summer of 2017, 34 *P. nobilis* specimens were sampled from three sites: Sv. Nedjelja (42°27′30.80″ N 18°40′26.70″ E; depth = 2 m; n = 14); Dobrota (42°26′13.50″ N 18°45′47.32″ E; depth = 5 m; n = 11) and Sv. Stasije (42°28′4.14″ N 18°45′44.28″ E; depth = 5 m; n = 9) within the Boka Kotorska Bay, Adriatic Sea, Montenegro (Figure 1). Sv. Nedjelja was used as the reference site due to the exceptionally high density of *P. nobilis* in this location [38], which indicates suitable environmental conditions for their development. Moreover, *P. nobilis* is a reliable bioindicator species for benthic coastal ecosystems [39]. Sv. Nedjelja is situated in the middle part of the bay, featured by a sandy bottom, a higher level of seawater exchange and lower salinity fluctuations during the year. *P. nobilis* population at this site was situated within the seagrass meadows of *Cymodocea nodosa* (Ucria) Aschers. The Dobrota and Sv. Stasije sites feature muddy sediments, a lower level of seawater exchange due to a higher inner positioning within the bay and higher seasonal salinity oscillations caused by freshwater inflow from the land and underwater springs. The seagrass communities were not developed at the Dobrota site while the *P. nobilis* population in Sv. Stasije was settled within the *Possidonia oceanica* (L.) Delile meadows. During the extraction of *P. nobilis* specimens from their natural habitat, it was important to avoid shell damage and maintain the byssus threads intact. This was achieved by a metal hand trowel which helped to maintain a certain amount of sediment around the buried part of the shell. The residual sediment was gently removed by hand to avoid seawater turbidity, since the specimens of *P. nobilis* were placed in tanks at 18 °C for transportation. Then, in 10–60 min, the fan mussels were transferred to the laboratory of the Institute of Marine Biology, Kotor, and placed in aquaria with clean seawater, constant aeration, temperature 20 ± 2 °C and salinity 29–36‰ for acclimation. The temperature and salinity were measured with a WTW Multi 350i probe (WTW GmbH; Weilheim, Germany), while the antero–posterior shell length and width of specimens were measured with an aluminum caliper. After laboratory manipulations (further in text) *P. nobilis* specimens were transplanted into the sediment at a 5 m depth and approximately 1 m distance between each other at the Dobrota site. It was important to bury them in sediment at least up to half of the shell in an appropriate orientation to minimize resistance to hydrodynamic forces. The whole extraction/transplantation procedure was conducted according to the protocol by [40,41]. 

### 2.2. Heart Rate Analyses

#### 2.2.1. Heart Rate Recording

**Figure 2:**
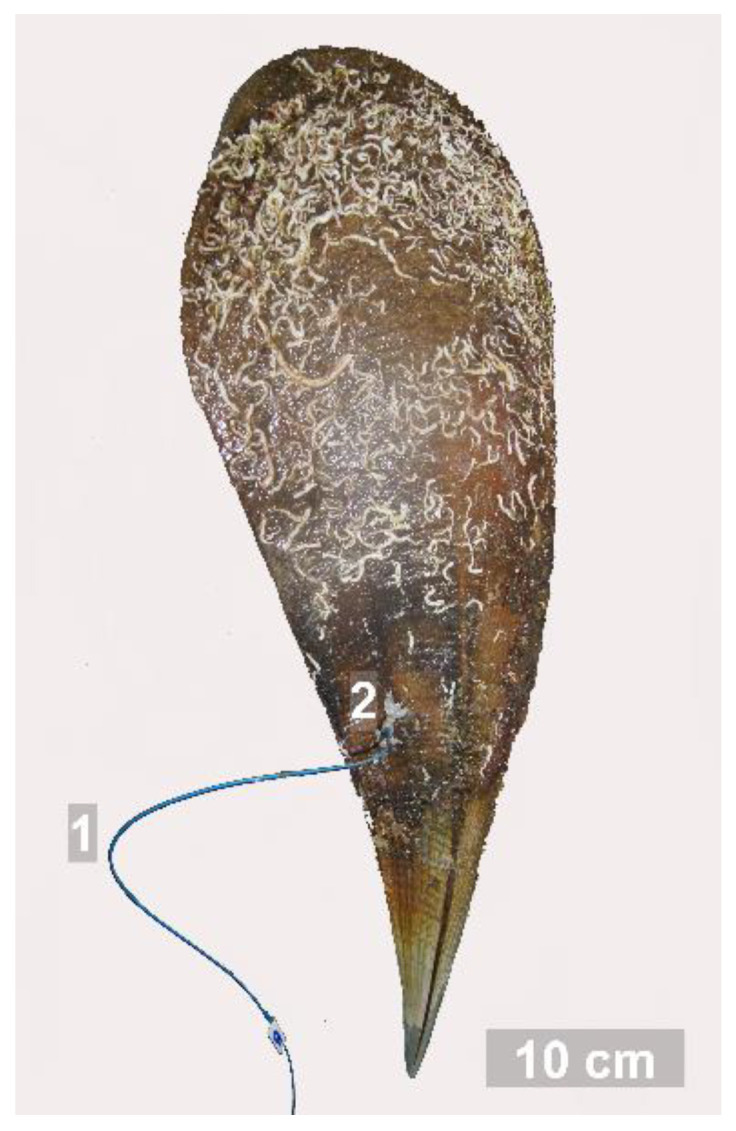
*Pinna nobilis* shell: 1—fiber-optic cable, 2—sensor attached above the heart area.

The Hr recording of the mussels was carried out by a non-invasive laser fiber-optic method developed in 1999 at the Research Center of Ecological Safety, Russian Academy of Sciences, St. Petersburg, Russia [11]. The applied method is based on photoplethysmography (PPG). PPG is a simple and inexpensive technology that includes a light source and a photodetector, often used for Hr monitoring [42]; it is focused on changes in light intensity reflected from or transmitted through the tissue, providing information on the heartbeats [43]. The whole procedure for the cardiac activity registration of benthic invertebrates was thoroughly explained by [15]. Briefly, the experimental unit includes eight PPG devices (Photoplethismograph, RIC “Eco-Contour”; Russia) allowing for the simultaneous recording of cardiac activity of eight mussels. *P. nobilis* specimens were polished by gentle sandpaper in a small region above the heart area to attach sensor holders by waterproof epoxy glue (Figure 2). The connection with the IR light source and receiver placed in the PPG device was made by fiber-optic cables. The sensor detected IR light which was reflected from the heart area and the data on periodical changes of the heart volume were transferred to a personal computer. After amplification and analog to digital conversion, the signal was processed by VarPulse 9.0 software (St. Peterburg, Russia), used for analyses of cardiac intervals [44].

#### 2.2.2. Hyposalinity Test

After the acclimation of the mussels, we carried out two experiments per site. For each one, 4–7 pen shell specimens were placed in seawater tanks for hyposalinity test, while one specimen per experiment in a separate aquarium was used as a control. Only one *P. nobilis* specimen was used as a control due to a lower number of specimens sampled at the two sites and the equipment limitation to eight animals per experiment. The standardized experimental procedure for marine species, which includes seawater salinity reduction, was conducted according to [12,15]. The hyposalinity test started by a gentle addition of distilled water to the seawater aquarium to reduce the salinity by 50%, as the ranges of the physiological tolerance limits for different species were defined previously [15]. Then, lower salinity values were measured, the salt solution was prepared based on calculation and added to the aquarium after approximately 1 h, to restore the background salinity determined prior to the test. Before the addition of distilled water, we recorded the Hr values in clean seawater for at least 3 h to establish normal Hr-resting response (Hr_rest_) in defined environmental conditions. Establishing a normal resting response is required in physiological assays due to considerable interindividual variability in physiological measurements [45].

#### 2.2.3. Calculations of T_rec_ and CV

The calculations of T_rec_ and CV were based on the result graphs of Hr pattern in MS Excel for each specimen separately (Figure S1) and presented as mean ± standard error within the groups of mussels from the sampling sites. T_rec_ was defined as the time–distance between two points (end of salinity restoration and re-achieving the stable Hr values recorded prior to the test), while CV was determined as the relation between standard deviation (SD) and mean value of Hr measured 1 h after the end of salinity restoration in the group of specimens for each experiment [16]. 

### 2.3. Statistical Analyses

Statistical analysis of the results obtained in six experiments was performed by Statistica 7.0 Software (StatSoft, Inc., Tulsa, OK, USA) [46]. The Kolmogorov–Smirnov test for normality of distribution was used prior to statistical analysis. Considering that the data were not in line with the requirements for the application of parametric tests, differences between each group and corresponding reference points were tested using the Mann–Whitney U test. Correlation analyses were carried out using the Spearman correlation test with a significance level *p* < 0.05.

## 3. Results 

**Figure 3:**
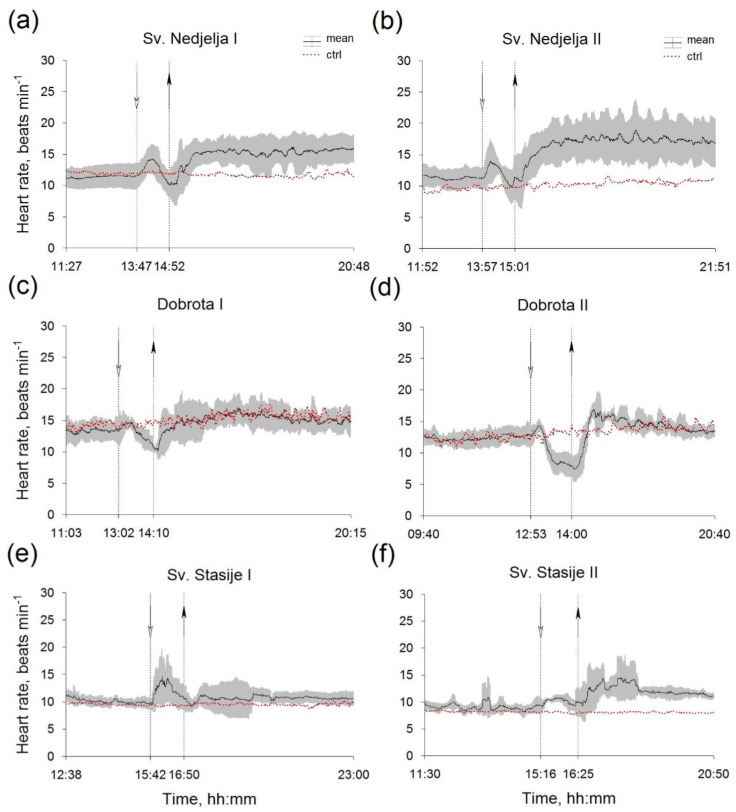
Mean HR within groups of *Pinna nobilis* specimens sampled from Sv. Nedjelja (**a**,**b**), Dobrota (**c**,**d**) and Sv. Stasije (**e**,**f**), before, during (between dashed lines) and after experimental treatment. Abbreviations: ctrl—control (red dotted lines); white head arrows—onset of salinity change by distilled water; black head arrows—onset of restoration of initial salinity values by salt addition; I—the first experiment; II—the second experiment.

The temperature and salinity of seawater in the aquaria, before, during and after the hyposalinity test are presented in Table 1, while the results for mean Hr of *P. nobilis* from all of the sampling sites are summarized in Figure 3. In all six experiments, the Hr of control specimens was stable by the end of recording (Figure 3). For both of the experiments within the groups of mussels from Sv. Nedjelja—reference site (Figure 3a,b) and from Dobrota (Figure 3a,b), mean Hr decreased by hyposalinity while the restoration of background salinity induced Hr elevation. On the other hand, in both of the experiments with mussels from Sv. Stasije (Figure 3e), hyposalinity caused an increase in the mean Hr, while in the second (Figure 3f), a mean Hr increase was observed after salinity restoration as well. In all of the experiments, immediately after hyposalinity onset, faster or slower, the mean Hr pattern showed a characteristic ridge shape (Figure 3), which is more visible on the Hr example of a single specimen (Figure S1). However, Hr of mussels showed interindividual variability within the groups, expressed as different responses during the hyposalinity test (SD, Figure 3). Regardless of their size, after initial Hr increase followed by decline during the lower salinity, in a few cases the Hr decrease did not occur. The observed Hr differences were statistically significant for each *P. nobilis* specimen in comparison between the periods before and during hyposalinity exposure. In the mussels sampled from Sv. Nedjelja, the mean T_rec_ (both experiments) was 72 ± 3 min, while in the mussels from Dobrota and Sv. Stasije, it was 91 ± 7 and 117 ± 15 min, respectively. Mean values of CV for both experiments within the groups of specimens from Sv. Nedjelja, Dobrota and Sv. Stasije were 0.12, 0.13 and 0.17, respectively.

**Figure 4:**
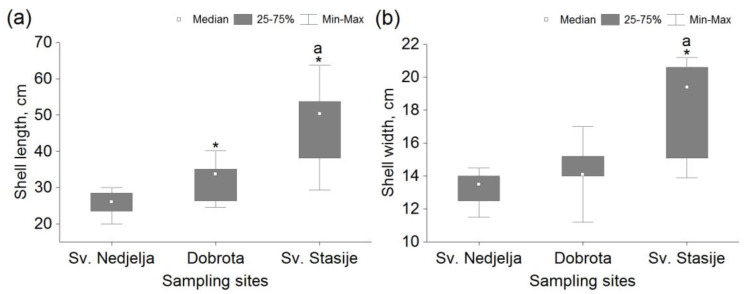
Antero–posterior shell length (**a**) and width (**b**) of *Pinna nobilis* sampled from Sv. Nedjelja (n = 12), Dobrota (n = 9) and Sv. Stasije (n = 7) within the Boka Kotorska Bay. *—statistically significant difference (*p* < 0.05) in comparison with the reference site (Sv. Nedjelja); a—statistically significant difference (*p* < 0.05) in comparison with the other sampling site; n—number of specimens.

There was no statistically significant difference (*p* > 0.05) of tested parameters in comparison between the two experiments carried out within the groups of mussels from the same sampling sites. The shell length of mussels from Dobrota and Sv. Stasije was significantly higher in comparison to Sv. Nedjelja, and the specimens from Sv. Stasije were significantly longer in comparison to Dobrota (Figure 4a). Statistically significant differences in shell width between the Sv. Nedjelja and Dobrota sites were not observed, while the specimens from Sv. Stasije were significantly wider in comparison to other sites (Figure 4b). 

**Figure 5:**
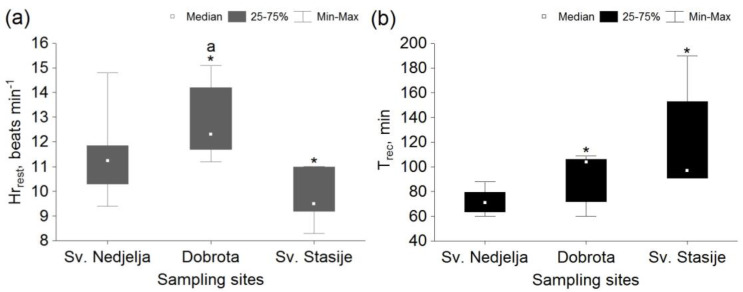
(**a**)—resting heart rate (Hr_rest_); (**b**)—heart rate recovery time (T_rec_) within a group of *Pinna nobilis* specimens sampled from Sv. Nedjelja, Dobrota and Sv. Stasije. *—statistically significant difference (*p* < 0.05) in comparison with the reference site (Sv. Nedjelja); a—statistically significant difference (*p* < 0.05) in comparison with the other sampling site.

Mean Hr_rest_ was significantly higher in mussels from Dobrota in comparison to other sites, while mussels from Sv. Stasije showed a significantly lower value of mean Hr_rest_ in comparison to Sv. Nedjelja (Figure 5a). There was no significant difference in T_rec_ between the specimens from the Dobrota and Sv. Stasije sites, however specimens from both sites showed significantly longer T_rec_ in comparison to Sv. Nedjelja (Figure 5b).

**Figure 6:**
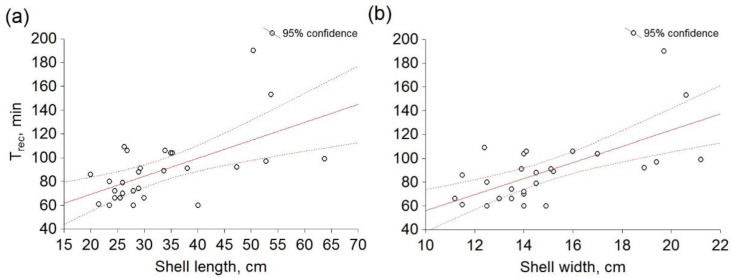
Relation between heart rate recovery time (T_rec_) and antero–posterior (**a**) shell length, (**b**) shell width of *Pinna nobilis*. Circles represent mean values obtained in six independent experiments.

Furthermore, a significant correlation (*p* < 0.05) was observed between the data for T_rec_ and shell length (*p* = 0.000528; r = 0.59; Figure 6a), and T_rec_ and shell width (*p* = 0.0000875; r = 0.65; Figure 6b). On the other hand, in comparison between Hr_rest_ and shell length, a significant correlation was not observed (*p* = 0.029318; r = −0.36; Figure S2a), nor between Hr_rest_ and shell width (*p* = 0.033571; r = −0.35; Figure S2b).

## 4. Discussion

If we compare the Hr pattern of *P. nobilis* in the control with control experiments of *M. galloprovincialis* (L.) [47,48], a lower Hr_rest_ and a very stable Hr of *P. nobilis* can be observed for a longer period without the Hr oscillations recorded in Mytilus. The reason could be the size difference between these two mollusk species, since the large shells of the freshwater mussel *Cristaria plicata* (Leach, 1815) showed lower mean values of Hr_rest_ [49]. Moreover, Hr of marine mussels *Perna viridis* (Linnaeus, 1758) [50] and *Chlamys* *farreri* (Jones et Preston, 1904) [51] showed a negative correlation with shell size. The reason for different shell sizes of *P. nobilis* specimens sampled from the three sites at the Boka Kotorska Bay was population structure. The Sv. Nedjelja and Sv. Stasije sites were mainly inhabited by younger/smaller and older/larger specimens, respectively, while at the Dobrota site, the population was comprised of small- to middle-sized specimens. In this study it was shown that the significantly longer and wider shells of *P. nobilis* specimens from Sv. Stasije have significantly lower Hr_rest_ in comparison to other sites. However, a significant correlation between the shell size and Hr_rest_ for all of the *P. nobilis* specimens was not observed, which supports our initial assumption to compare Hr within the groups of mussels with different shell sizes. Furthermore, the gradual Hr increase in *P. nobilis* at the onset of the hyposalinity test (ridge-shaped Hr pattern) in comparison to the faster response of Mytilus [13,15] could be explained by the larger volume of seawater with higher salinity which remains for longer inside the large shell of *P. nobilis* upon the valve closure. Isolation response to environmental salinity reduction by closing shell valves accompanied with Hr decrease occurs due to the restriction of the gas exchange and aerobic metabolism [52]. In a few cases, the Hr of *P. nobilis* showed only the increase phase, despite the fact that a further decline in Hr value was expected for marine species based on the hyposalinity tests in the aforementioned studies. The observed Hr differences between the *P. nobilis* specimens contributed to interindividual variability as the response to hyposalinity conditions. Thus, it would be recommendable to prolong the duration of the hyposalinity test to 1.5–2 h to have higher uniformity in the Hr response for this species and probably others with a larger shell size as well. 

Significantly shorter T_rec_ of the *P. nobilis* specimens from the reference site in comparison with Dobrota and Sv. Stasije showed a higher adaptive capacity and good health condition which is in compliance with our premise of a good ecological status at this site featured by a very high density of *P. nobilis* population [38]. In addition, the good health state of rock crabs from clean sites was confirmed by Hr and other physiological parameters [53]. In other hyposalinity or hypersalinity studies performed on different marine and freshwater mollusks [13,15,16,18], the T_rec_ values for the reference sites were in a similar range as the T_rec_ of *P. nobilis* measured in this study. According to recommendations [12], the specimens from Dobrota and Sv. Stasije with significantly longer T_rec_ in comparison with the reference site had a lower compensatory response to stress probably due to poor health condition. Considering the close relationship between *P. nobilis* and the sea bottom, the higher content of certain trace elements detected in the sediments at the Dobrota site [54], and the increased lead concentration in seawater, sediments and the seagrass *P. oceanica* found at Sv. Stasije [55] could contribute to the accumulation of these contaminants in the fan mussels’ tissues and affect their physiology. Particularly, trace element contamination in the tissues of the Mediterranean mussels from the Boka Kotorska Bay induced longer T_rec_ after the hyposalinity test [16]. In this study, we have not performed any toxicological analyses due to the existing sediment data of the investigated sites and to avoid tissue sampling of endangered species, such as *P. nobilis*. However, the lower adaptive capacity, defined as the poor health state [12,15], of *P. nobilis* specimens indicated by Hr response could be caused by deterioration of environmental conditions at the studied sites.

On the other hand, the specimens from all of the three sites showed very low CV values specific for healthy individuals inhabiting clean environments which indicated that the differences in the health status of these individuals are not dramatic. In general, the significant correlation between T_rec_ and the shell size of *P. nobilis* is in compliance with data on CV. However, despite the significant difference in the shell size between the specimens from Dobrota and Sv. Stasije, a significant difference in T_rec_ was not observed. Accordingly, these data showed that the significantly longer T_rec_ of the specimens from the studied sites in comparison to the reference site was not a consequence of shell size.

As far as we know, these are the first data on the Hr of *P. nobilis*. Based on our experience, *P. nobilis* is a very suitable model organism for studying cardiac activity by non-invasive PPG method. Despite its vulnerability in nature, *P. nobilis* showed a sufficient level of resilience during handling. Moreover, the *P. nobilis* shell is firm, flat and thin above the heart area which contributes to more precise sensor positioning and better Hr signal transduction. Furthermore, based on the results of this study, the most appropriate size of *P. nobilis* specimens for Hr monitoring purposes belongs to the size class, 30–40 cm. The smaller *P. nobilis* individuals needed more time for shell surface preparation due to densely spaced spines near the heart region, while the larger individuals were less suitable for handling since these shells needed more space and larger amounts of seawater for maintenance in the laboratory tanks. 

After laboratory analyses, the specimens were transplanted in a more distant environment from the open sea with a lower level of seawater exchange at the inner part of the Boka Kotorska Bay on the Dobrota site due to the possibility of *P. nobilis* MME spreading from the western Mediterranean [24] at that time. Another reason in favor of this decision was the unhindered growth of the *P. nobilis* population from Dobrota under a lower salinity regime [56], knowing that salinity is a limitation factor for the spread of *P. nobilis* MME [27]. It was also beneficial to select an environment already inhabited by *P. nobilis* to increase transplantation success [40]. All the specimens survived for at least two years, until Spring 2019 and *P. nobilis* MME appeared in the Montenegrin coast (our unpublished data), which indicates a well-performed transplantation. 

*P. nobilis* Hr, featured by specific response to stress, could be suggested as a potential biomarker of distress, such as marine pollution or poor ex situ maintenance conditions of individuals maintained indoors. The experimental unit used in this study is particularly suitable for this purpose, because it is capable of real-time observation of cardiac activity in 10 s intervals and continuous long-term registration [44]. By using this Hr recording methodology, it is also possible to reveal early signs of physiological impairment in rescued individuals to quickly undertake the measures needed for survival in captivity. The method could be used to separate diseased and healthy individuals, which would not show external symptoms of disease in the initial phases of the infection, reducing the possibilities of cross-infections and increasing the chances of survival of uninfected individuals [57]. This is especially so since similar sensor technology applied on blue mussels indicated Hr changes as the response to *Himasthla elongata* (Mehlis, 1831) parasite infection [58]. More studies should be completed in this regard, to differentiate Hr between healthy and infected individuals by *H. pinnae*. The method is also easily adaptable to monitor other commercial and non-commercial species of sessile invertebrates to check their health status. 

Thus, investigation focused on the maintenance of rescued individuals in captivity should be prioritized, studying their adaptive capacity under physico-chemical parameters variations by means of non-invasive techniques. It could be important for future repopulation trials in order to make the right decisions on the selection of environments suitable for the growth of *P. nobilis* but constrained or restricted for pathogen development. Moreover, an earlier long-term study [59] found that hyposalinity suppressed the distribution of a haplosporidian parasite in oyster beds. The same was observed in the case of *P. nobilis* within the littoral lagoons and deltas of Spain and France in the aforementioned studies. Accordingly, the ex situ monitoring of *P. nobilis*‘ physiological state under short and intensive hyposalinity could be important to obtain new insights of species’ salinity tolerance toward development of potential prophylactic treatment against *H. pinnae*, needed for the survival of already infected specimens, since [57] reported some positive responses to temperature and salinity treatments of the *P. nobilis* in captivity infected by *H. pinnae*.

## Figures and Tables

**Table 1:** Seawater temperature and salinity in aquaria with *Pinna nobilis* specimens sampled from the sites Sv. Nedelja, Dobrota and Sv. Stasije, measured before hyposalinity test (background), during the 50% reduction by distilled water (salinity reduction) and after the restoration by salt addition (salinity restoration). Abbreviations: Exp.—experiment; Temp.—temperature.

Sampling Sites	Exp. No.	Temp. (°C)	Background Salinity (‰)	Salinity Reduction (‰)	Salinity Restoration (‰)
Sv. Nedjelja	I	19.4	36	19.2	36.3
	II	20.3	34.8	18.3	35.6
Dobrota	I	20.1	29.3	15	30
	II	22.3	35.3	17.8	35
Sv. Stasije	I	19.5	32.4	17.1	33.1
	II	21.4	33.5	16.6	33

## Data Availability

Not applicable.

## Supplementary Materials

The following supporting information can be downloaded at: https://www.mdpi.com/article/10.3390/mi13091549/s1, Figure S1: Example of the heart rate pattern of pen shell *P. nobilis* in clean sea water (Hr_rest_), during ≈ 1 h of 50% salinity reduction by distilled water (dH_2_O) and after salt addition. Abbreviations: T_rec_—heart rate recovery time; Figure S2: Relation between resting heart rate (Hr_rest_) and antero–posterior (a) shell length, (b) shell width of *P. nobilis*. Circles represent mean values obtained in six independent experiments.
