# Supplementary material for: Evaluation of Physiological State of Pen Shell Pinna nobilis (Linnaeus, 1758) by a Non-Invasive Heart Rate Recording under Short-Term Hyposalinity Test
Source: Micromachines (Basel). 2022 Sep 18;13(9):1549. doi: 10.3390/mi13091549 (PMC9505886; doi:10.3390/mi13091549)
Supplement: Supplementary file 1 [file micromachines-13-01549-s001.zip › micromachines-1900425-supplementary.pdf]

micromachines

MDPI

Supplementary files:

Evaluation of Physiological State of Pen Shell *Pinna nobilis* (Linnaeus, 1758) by a Non-Invasive Heart Rate Recording under Short-Term Hyposalinity Test

Rajko Martinović, Danijela Joksimović, José Rafael García-March, Nardo Vicente and Zoran Gačić

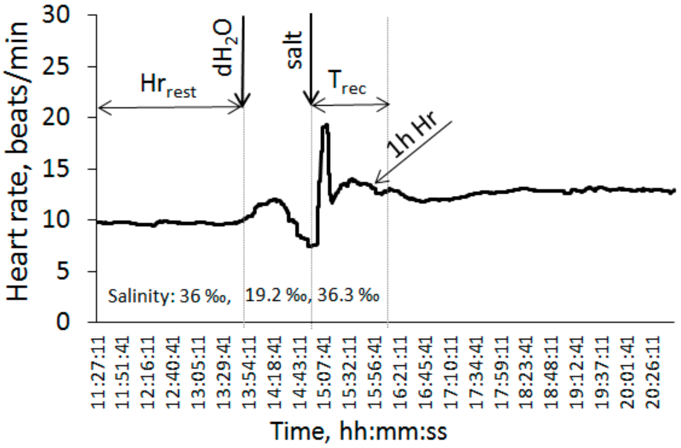

Figure S1. Example of the heart rate pattern of pen shell *P. nobilis* in clean sea water (Hr_{rest}), during ≈ 1h of 50 % salinity reduction by distilled water (dH_2O) and after salt addition. Abbreviations: T_{rec} – heart rate recovery time.

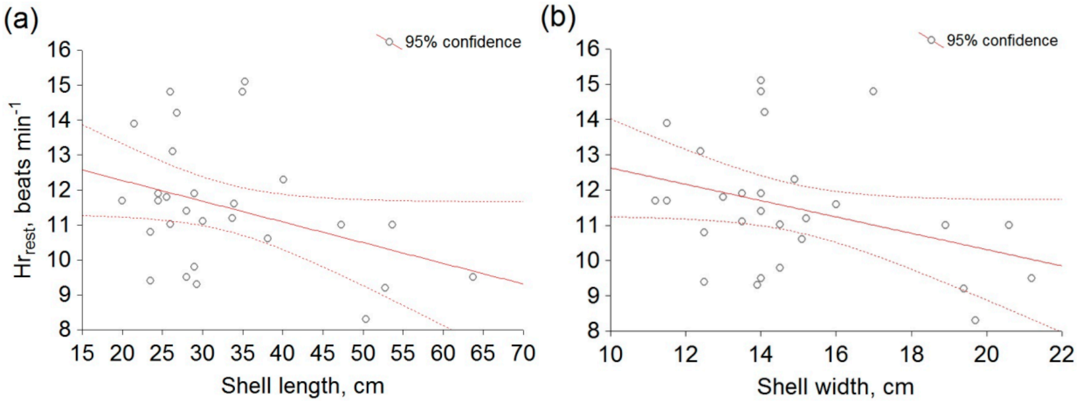

Figure S2. Relation between resting heart rate (Hr_{rest}) and antero-posterior (a) shell length, (b) shell width of *P. nobilis*. Circles represent mean values obtained in six independent experiments.
